# Draft Genome Sequence of *Pyrodictium occultum* PL19^T^, a Marine Hyperthermophilic Species of *Archaea* That Grows Optimally at 105°C

**DOI:** 10.1128/genomeA.00016-16

**Published:** 2016-02-25

**Authors:** Sagar M. Utturkar, Harald Huber, Sebastian Leptihn, Belinda Loh, Steven D. Brown, Karl O. Stetter, Mircea Podar

**Affiliations:** a Biosciences Division, Oak Ridge National Laboratory, Oak Ridge, Tennessee, USA; b Graduate School of Genome Science and Technology, University of Tennessee, Knoxville, Tennessee, USA; c Lehrstuhl fur Mikrobiologie und Archaeenzentrum, Universitaet Regensburg, Regensburg, Germany; d Institute for Microbiology and Molecular Biology, University of Hohenheim, Stuttgart, Germany

## Abstract

We report here the draft genome sequence of *Pyrodictium occultum* PL19^T^, a marine hyperthermophilic archaeon. The genome provides insights into molecular and cellular adaptation mechanisms to life in extreme environments and the evolution of early organisms on Earth.

## GENOME ANNOUNCEMENT

*Pyrodictium occultum* PL19^T^, a member of the order *Desulfurococcales* (phylum *Crenarchaeota*, kingdom *Archaea*), was the first hyperthermophile cultured in the laboratory at temperatures above the boiling point of water (1). Isolated from a shallow submarine solfataric field near the Volcano Island (Italy), *P. occultum* has an optimum growth temperature of 105°C and is an obligate chemoautotrophic anaerobe, which fixes CO_2_ using energy derived from sulfur reduction by hydrogen (2). These characteristics represent adaptations to physical and chemical conditions associated with early Earth environments and may indicate that such organisms are ancient survivors from billions of years ago (3). Life at such high temperatures poses unique biochemical adaptation challenges in terms of macromolecular stabilization and biosynthesis of heat-labile metabolites. *P. occultum* and subsequently isolated related species were used for some of the first molecular characterizations of such adaptations, including the presence of abundant specialized chaperones, nucleotide modifications, and enzymatic activities of individually isolated proteins and energy-generating membrane-associated complexes at boiling temperatures (4–10).

*P. occultum* PL19^T^ was cultured anaerobically at 105°C in artificial seawater medium, with H_2_ as the energy source (2), at the Archaeenzentrum facility of the University of Regensburg (Germany). Genomic sequencing was performed using the Illumina MiSeq platform, followed by quality-based read trimming, as described previously (11), which generated 22.5 million paired-end reads, with an average length of 276 bp. After evaluating several approaches (12), the optimal assembly statistics were obtained using the SPAdes software (version 3.1.0) (13) and consisted of 14 large (≥500 bp) contigs. After additional read mapping to extend the contig ends and superassembly with Geneious (version 8.1) (14), we obtained a final assembly composed of 2 contigs, with a total genome size of 1.6 Mbp. Gene prediction and annotation were performed using NCBI Prokaryotic Genome Automatic Annotation Pipeline (PGAAP) (15). The draft genome has an overall G+C content of 63.4%, a largest contig of 1.5 Mbp, and 3,360 candidate protein-coding genes. Genome integrity was confirmed by the CheckM tool (16), which estimated 98.1% completeness based on presence of 245 marker genes.

A unique characteristic of *Pyrodictium* species is the formation of an extracellular network of thin filaments, tens of micrometers long, which connect many cells in millimeter-sized mycelium-like aggregates (1, 17). The filaments are composed of hollow ultrathin (~30-nm diameter) tubes (cannulae) that penetrate the periplasmic space of individual cells and assemble from at least three related proteins, characterized biochemically from *Pyrococcus abyssi* (18, 19). The biological function of those tubes has remained unknown for decades, and no sequence or structural homologues of the cannula proteins can be identified in any other organisms. In the genome of *P. occultum*, we identified five genes likely encoding cannula proteins (CF15_01655, CF15_06945, CF15_06965, CF15_07030, and CF15_07065). The availability of the *P. occultum* genome sequence should further enable evolutionary, physiological, and molecular investigations of these hyperthermophilic archaea.

### Nucleotide sequence accession numbers.

This whole-genome shotgun project has been deposited at DDBJ/EMBL/GenBank under the accession no. LNTB00000000. The version described in this paper is version LNTB01000000.

## References

[1] Stetter KO 1982 Ultrathin mycelia-forming organisms from submarine volcanic areas having an optimum growth temperature of 105 °C. Nature 300:258–260. doi:10.1038/300258a0.

[2] Stetter KO, König H, Stackebrandt E 1983 *Pyrodictium* gen. nov., a new genus of submarine disc-shaped sulphur reducing archaebacteria growing optimally at 105°C. Syst Appl Microbiol 4:535–551. doi:10.1016/S0723-2020(83)80011-3. 23194811

[3] Stetter KO 2006 Hyperthermophiles in the history of life. Philos Trans R Soc Lond B Biol Sci 361:1837–1843. 17008222 10.1098/rstb.2006.1907 PMC1664684

[4] Dirmeier R, Hauska G, Stetter KO 2000 ATP synthesis at 100°C by an ATPase purified from the hyperthermophilic archaeon *Pyrodictium abyssi*. FEBS Lett 467:101–104. doi:10.1016/S0014-5793(00)01131-5. 10664465

[5] Keller M, Dirmeier R 2001 Hydrogen-sulfur oxidoreductase complex from *Pyrodictium abyssi*. Methods Enzymol 331:442–451. 11265482 10.1016/s0076-6879(01)31075-3

[6] Minuth T, Frey G, Lindner P, Rachel R, Stetter KO, Jaenicke R 1998 Recombinant homo- and hetero-oligomers of an ultrastable chaperonin from the archaeon *Pyrodictium occultum* show chaperone activity *in vitro*. Eur J Biochem 258:837–845. doi:10.1046/j.1432-1327.1998.2580837.x. 9874254

[7] Parameswaran AK, Provan CN, Sturm FJ, Kelly RM 1987 Sulfur reduction by the extremely thermophilic archaebacterium *Pyrodictium occultum*. Appl Environ Microbiol 53:1690–1693. 16347396 10.1128/aem.53.7.1690-1693.1987 PMC203932

[8] Takeda N, Nakamura M, Yoshizumi H, Tatematsu A 1994 Detection of ribose-methylated nucleotides in *Pyrodictium occultum* tRNA by liquid chromatography—frit-fast atom bombardment mass spectrometry. J Chromatogr B Biomed Appl 660:223–233. doi:10.1016/0378-4347(94)00299-1. 7866511

[9] Dirmeier R, Keller M, Frey G, Huber H, Stetter KO 1998 Purification and properties of an extremely thermostable membrane-bound sulfur-reducing complex from the hyperthermophilic *Pyrodictium abyssi*. Eur J Biochem 252:486–491. doi:10.1046/j.1432-1327.1998.2520486.x. 9546664

[10] Pihl TD, Schicho RN, Kelly RM, Maier RJ 1989 Characterization of hydrogen-uptake activity in the hyperthermophile *Pyrodictium brockii*. Proc Natl Acad Sci USA 86:138–141. doi:10.1073/pnas.86.1.138. 2492097 PMC286419

[11] Utturkar SM, Klingeman DM, Bruno-Barcena JM, Chinn MS, Grunden AM, Köpke M, Brown SD 2015 Sequence data for *Clostridium autoethanogenum* using three generations of sequencing technologies. Sci Data 2:150014. doi:10.1038/sdata.2015.14. 25977818 PMC4409012

[12] Utturkar SM, Klingeman DM, Land ML, Schadt CW, Doktycz MJ, Pelletier DA, Brown SD 2014 Evaluation and validation of *de novo* and hybrid assembly techniques to derive high quality genome sequences. Bioinformatics 30:2709–2716 doi:10.1093/bioinformatics/btu391. 24930142 PMC4173024

[13] Bankevich A, Nurk S, Antipov D, Gurevich AA, Dvorkin M, Kulikov AS, Lesin VM, Nikolenko SI, Pham S, Prjibelski AD, Pyshkin AV, Sirotkin AV, Vyahhi N, Tesler G, Alekseyev MA, Pevzner PA 2012 SPAdes: a new genome assembly algorithm and its applications to single-cell sequencing. J Comput Biol 19:455–477. doi:10.1089/cmb.2012.0021. 22506599 PMC3342519

[14] Kearse M, Moir R, Wilson A, Stones-Havas S, Cheung M, Sturrock S, Buxton S, Cooper A, Markowitz S, Duran C, Thierer T, Ashton B, Meintjes P, Drummond A 2012 Geneious basic: an integrated and extendable desktop software platform for the organization and analysis of sequence data. Bioinformatics 28:1647–1649. doi:10.1093/bioinformatics/bts199. 22543367 PMC3371832

[15] Angiuoli SV, Gussman A, Klimke W, Cochrane G, Field D, Garrity G, Kodira CD, Kyrpides N, Madupu R, Markowitz V, Tatusova T, Thomson N, White O 2008 Toward an online repository of Standard Operating Procedures (SOPs) for (meta)genomic annotation. Omics 12:137–141. doi:10.1089/omi.2008.0017. 18416670 PMC3196215

[16] Parks DH, Imelfort M, Skennerton CT, Hugenholtz P, Tyson GW 2015 CheckM: assessing the quality of microbial genomes recovered from isolates, single cells, and metagenomes. Genome Res 25:1043–1055. doi:10.1101/gr.186072.114. 25977477 PMC4484387

[17] Rieger G, Rachel R, Hermann R, Stetter KO 1995 Ultrastructure of the hyperthermophilic archaeon *Pyrodictium abyssi*. J Struct Biol 115:78–87. doi:10.1006/jsbi.1995.1032.

[18] Nickell S, Hegerl R, Baumeister W, Rachel R 2003 *Pyrodictium* cannulae enter the periplasmic space but do not enter the cytoplasm, as revealed by cryo-electron tomography. J Struct Biol 141:34–42. doi:10.1016/S1047-8477(02)00581-6. 12576018

[19] Mai B 1998 *In-vitro-*untersuchungen zum extrazellularen netzwerk von *Pyrodictium abyssi* TAG11. University of Regensburg, Regensburg, Germany.

